# A Fluorescence Reporter Model Defines “Tip-DCs” as the Cellular Source of Interferon β in Murine *Listeriosis*

**DOI:** 10.1371/journal.pone.0015567

**Published:** 2010-12-16

**Authors:** Philipp Dresing, Stephanie Borkens, Magdalena Kocur, Sonja Kropp, Stefanie Scheu

**Affiliations:** Institute of Medical Microbiology and Hospital Hygiene, University of Düsseldorf, Düsseldorf, Germany; University of California San Francisco, United States of America

## Abstract

Production of type I interferons, consisting mainly of multiple IFNα subtypes and IFNβ, represents an essential part of the innate immune defense against invading pathogens. While in most situations, namely viral infections, this class of cytokines is indispensable for host survival they mediate a detrimental effect during infection with *L. monocytogenes* by rendering macrophages insensitive towards IFNγ signalling which leads to a lethal bacterial pathology in mice. Due to a lack of suitable analytic tools the precise identity of the cell population responsible for type I IFN production remains ill-defined and so far these cells have been described to be macrophages. As in general IFNβ is the first type I interferon to be produced, we took advantage of an IFNβ fluorescence reporter-knockin mouse model in which YFP is expressed from a bicistronic mRNA linked by an IRES to the endogenous *ifnb* mRNA to assess the IFNβ production on a single cell level *in situ*. Our results showed highest frequencies and absolute numbers of IFNβ^+^ cells in the spleen 24 h after infection with *L. monocytogenes* where they were located predominately in the white pulp within the foci of infection. Detailed FACS surface marker analyses, intracellular cytokine stainings and T cell proliferation assays revealed that the IFNβ^+^ cells were a phenotypically and functionally further specialized subpopulation of TNF and iNOS producing DCs (Tip-DCs) which are known to be essential for the early containment of *L. monocytogenes* infection. We proved that the IFNβ^+^ cells exhibited the hallmark characteristics of Tip-DCs as they produced iNOS and TNF and possessed T cell priming abilities. These results point to a yet unappreciated ambiguous role for a multi-effector, IFNβ producing subpopulation of Tip-DCs in controlling the balance between containment of *L. monocytogenes* infection and effects detrimental to the host driven by IFNβ.

## Introduction

*Listeria monocytogenes* is a Gram positive foodborne bacterial pathogen with a facultative intracellular life cycle that is widely used as a model organism to study the mammalian innate and adaptive immune response to infections [1], [2]. During systemic dissemination *L. monocytogenes* mainly replicates within cells of the spleen and the liver. After cellular invasion of the host cell the bacterium first resides within the phagosome. Due to expression of the *hyl* encoded pore forming hemolysin listeriolysin O (LLO) *L. monocytogenes* escapes from this hostile environment by disrupting the phagosomal membrane. The invasion of the cytoplasm is the basis for both the induction of innate response and long term protective immunity.

Cytosolic invasive *L. monocytogenes* are detected by a so far not identified cytoplasmic receptor that induces expression of type I IFNs [3]. The family of type I IFNs comprise of a single IFNβ and over a dozen IFNαs and share the same type I IFN receptor (IFNAR) [4]. While in virus infections type I IFNs in general protect the host, they play a more ambiguous role in bacterial infections [5]. Mice deficient for IFNAR, IFNβ or interferon regulatory factor 3 (IRF3) are less susceptible to *L. monocytogenes* infection compared to wt mice [6]–[9]. Multiple reasons for this effect were supposed. Type I IFNs sensitize T cells to apoptosis as they enhance the toxic effect of LLO. Macrophages phagocytising dying T cells produce anti inflammatory IL-10 that dampens inflammation. As IFNAR lacking mice possess higher frequencies of TNF producing cells type I IFNs contribute to the diminishment of essential effector cells necessary for bacterial clearance [10]. Recently one additional important mechanism for this effect was unravelled. Type I IFNs released from *L. monocytogenes* infected cells induce the downregulation of the IFNγ receptor and in this way renders the host refractory to IFNγ, a cytokine crucial for host resistance to *L. monocytogenes* [11]. Recruitment of monocytes is a further essential pillar of innate defence in listeriosis. Circulating monocytes are very plastic immune effector cells that act as precursors for several tissues macrophage subsets or give rise to dendritic cells (DCs) [12], [13]. Based on the differential expression of Ly6C monocytes can be divided into Ly6C^hi^ inflammatory monocytes and Ly6C^low^ monocytes that exhibit a crawling phenotype and patrol the vascular endothelium [14]. After i.p. infection with *L. monocytogenes* Ly6C^low^ monocytes rapidly extravasate into the peritoneum, induce an early inflammatory response by secretion of TNF, and activate genes involved in macrophage differentiation. In contrast to this, Ly6C^hi^ inflammatory monocytes are recruited to inflamed tissues and lymphnodes and are able to differentiate into inflammatory DCs [14]–[16]. After systemic *L. monocytogenes* challenge inflammatory monocytes are recruited to the spleen and give rise to TNF and iNOS producing DCs (Tip-DCs). Tip-DCs are CD11b^+^, Ly6C^hi^, Mac-3^hi^ and express intermediate levels of CD11c. They are essential sources of TNF and nitric oxide and crucial for the early containment of the bacterial growth after *L. monocytogenes* infection [16].

Since the expression of type I IFNs is detrimental during *L. monocytogenes* infection it is crucial to characterize the cells responsible for its production. As IFNβ is the type I IFN produced first in the majority of cases it is important to gain insights into which cell types are accountable for its expression and where they are located within the infected organism. To identify IFNβ producing cells we make use of an IFNβ/YFP reporter mouse (IFNβ^mob/mob^, mob: messenger of IFNβ) which expresses YFP from a bicistronic mRNA linked by an IRES to the endogenous IFNβ message [17]. In this paper we show that the vast majority of IFNβ producing cells were located within the foci of infection in the splenic white pulp. Detailed analysis of the surface and functional phenotype of the IFNβ producing cells reveals that they were a further specialized sub population of Tip-DCs.

## Results

### The spleen is the major site of the *L. monocytogenes* induced IFNβ response

**Figure 1:**
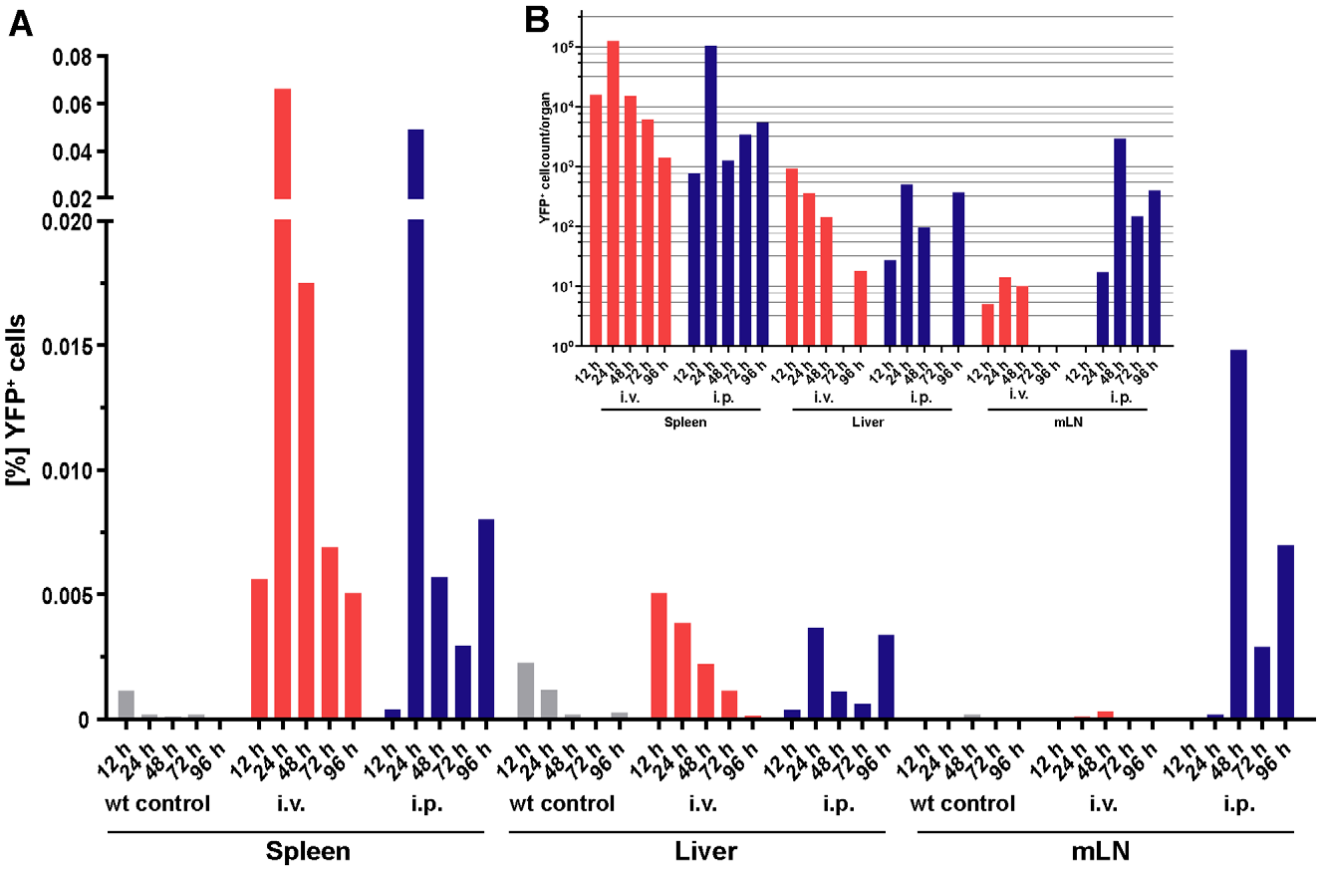
*L. monocytogenes* challenge induces highest numbers of IFNβ/YFP^+^ cells 24 hpi. in the the spleen. IFNβ^mob/mob^ or wt mice were infected with sublethal doses of *L. monocytogenes* as determined by LD_50_ survival experiments for the stated timepoints and route of infection (data not shown). The bars show the percentages (A) and absolute numbers (B) of YFP^+^ cells as measured by flow cytometry in the respective organs. The data shown is from at least two independent experiments with two mice per timepoint.

To quantify the IFNβ producing cells *in vivo* we infected IFNβ^mob/mob^ mice, that carry an IRES driven *yfp* reporter cassette linked to the endogenous *ifnb* locus i.v. or i.p. with *L. monocytogenes* and monitored the time course of IFNβ expression in the spleen, the liver and the mesenteric lymph nodes (mLNs) by flow cytometry (Figure 1). Regardless of the route of infection we observed the highest frequencies and maximal total numbers of YFP^+^ cells of all organs 24 h post infection (hpi.) in the spleen with about 0,06% of all splenocytes. This equals 10^5^ cells per spleen on average (Figure 1B). As in the spleen we could detect YFP^+^ cells in the liver after both i.v. and i.p. infection, but even at the peak of YFP expression after 12 to 24 hpi. the total IFNβ/YFP^+^ cell count was with ∼10^3^ cells per organ 100 fold lower as compared to the spleen. Due to massive hepatocyte cell death during sample preparation we decided to include only the leukocyte fraction of the liver in these analyses. In the mLNs 0.015% of the analysed cells were YFP^+^ 48 hpi. after i.p. infection whereas investigation after i.v. challenge didn't reveal a significant number of IFNβ/YFP^+^ cells. This indicates a dependency of IFNβ expression on the route of infection in the mLNs. To elucidate a possible correlation of the cellular IFNβ response in the spleen and titrated infection doses of *L. monocytogenes*, 10^5^ to 10^7^ CFU were injected i.v. into IFNβ^mob/mob^ mice. This resulted in a dose dependent increase of the bacterial load in the spleen while the absolute numbers of YFP^+^ cells peaked after infection with 10^6^ CFU of *L. monocytogenes* and did not further increase after injection of 10^7^ CFU (Figure S1). In summary, these data show that the spleen acts as the major site of IFNβ production during *L. monocytogenes* infection with as few as 10^5^ cells at the peak of the response being responsible for the production of biologically effective amounts of this cytokine.

### IFNβ producing cells in murine listeriosis exhibit an activated inflammatory monocyte phenotype

**Figure 2:**
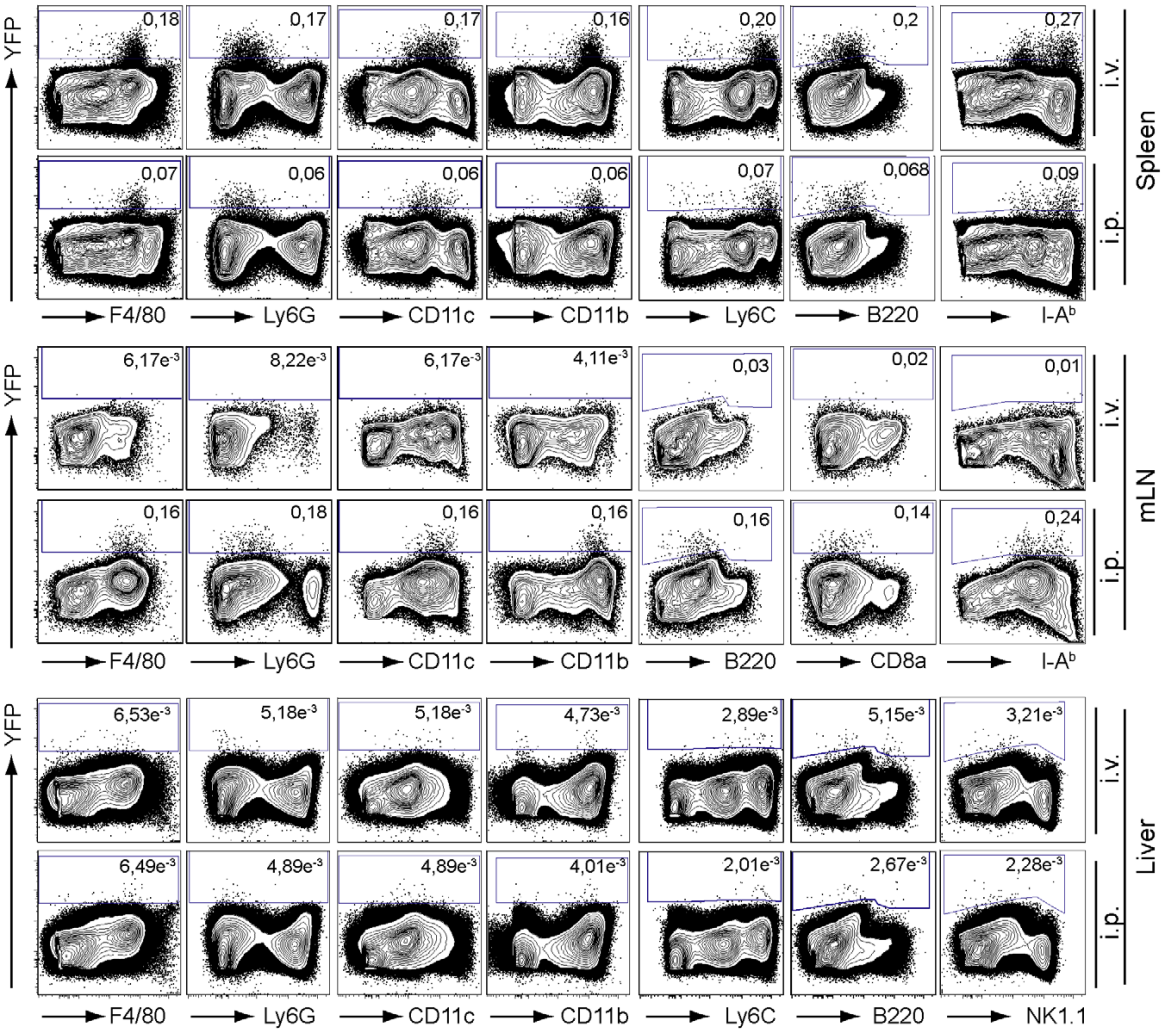
Peak frequencies of IFNβ expressing cells after *L. monocytogenes* infection in spleen, liver and mLNs. The peaks of IFNβ production were determined by timecourse experiments and infection dose titrations for spleen, mLNs and liver, respectively. IFNβ/YFP^+^ cells from the spleen and the liver are shown 24 h after i.v. infection with 10^6^ or i.p. infection with 10^7^ CFU of *L. monocytogenes*. IFNβ producing cells in the mLNs are presented 48 h after i.v. infection with 10^5^ or i.p. infection with 10^6^ CFU of *L. monocytogenes*. The cell populations were electronically gated on CD19^−^ CD3ε^−^ live cells. YFP gating was adjusted to equally treated wt references (not shown). The data shown is representative for at least two independent experiments with two mice per timepoint.

Appling direct *ex vivo* FACS characterization we observed that the vast majority of YFP^+^ cells after *L. monocytogenes* infection in spleen, liver and mLNs were negative for B220, Ly6G and NK1.1 ruling out the a potential contribution of plasmacytoid DCs, granulocytes, and NK cells to IFNβ production (Figure 2 and data not shown). In contrast, IFNβ producing cells were positive for F4/80, MHC class II, CD11b and show a high expression level of Ly6C and the activation markers CD40, CD80, CD86, and CD69 (Figure 2, Figure S2). This surface phenotype of the YFP^+^ cells suggests that they are a subpopulation of activated inflammatory monocytes [12], [18]. The YFP^+^ cells show an intermediate expression of CD11c, the classical marker expressed on dendritic cells (DCs). As inflammatory monocytes can differentiate to either macrophages or DCs [19] we recapitulated these potential developmental capacities *in vitro* by differentiating bone marrow cells into DCs (GM-CSF DCs, FLT3-L DCs) or macrophages (BMDMs), respectively. The derived cell types were then infected with the wt *L. monocytogenes* strain or a mutant strain deficient for *hly*, respectively. The Δ*hly* strain of *L. monocytogenes* is deficient for the *hly* encoded listeriolysin O (LLO). Therefore these bacteria cannot escape from the phagosome and are readily eliminated without inducing a type I IFN response [6], [20]. Wt *L. monocytogenes* induced IFNβ/YFP expression in a subpopulation of BMDMs (Figure S3A). Infection of GMCSF-DCs and FLT-3L derived DCs with wt *L. monocytogenes* likewise resulted in the expression of IFNβ/YFP from a small subset of DCs (Figure S3B). In contrast to this and in accordance with previous data, IFNβ/YFP^+^ cells were barely detectable in both DCs and BMDMs after challenge with Δ*hly* bacteria (Figure S3). As a marked exception, we observed in the liver in addition to the canonical myeloid IFNβ/YFP^+^ population a minor subpopulation of YFP^+^ cells that were positive for B220, Ly6C and CD11c and show the typical phenotype of pDCs. This result is reflected in *L. monocytogenes* infected FLT3-L DC cultures were B220^+^ cells, the *in vitro* analogue to pDCs, exhibit IFNβ/YFP production. In conclusion, YFP was produced from both BMDMs and GMCSF-DCs myeloid lineage derived cells *in vitro* which recapitulates the differentiation potential of inflammatory monocytes we found accountable for the IFNβ production after bacterial challenge *in vivo*. This hints at activated inflammatory monocytes being the major producers of IFNβ during *L. monocytogenes* Infection.

As the spleen turned out to be the major dwelling place of IFNβ producing cells after *L. monocytogenes* infection we focused our following *in vivo* experiments on the splenic IFNβ expressing cells.

### IFNβ producing cells in the spleen are located within the foci of infection and predominantly contain *L. monocytogenes*

**Figure 3:**
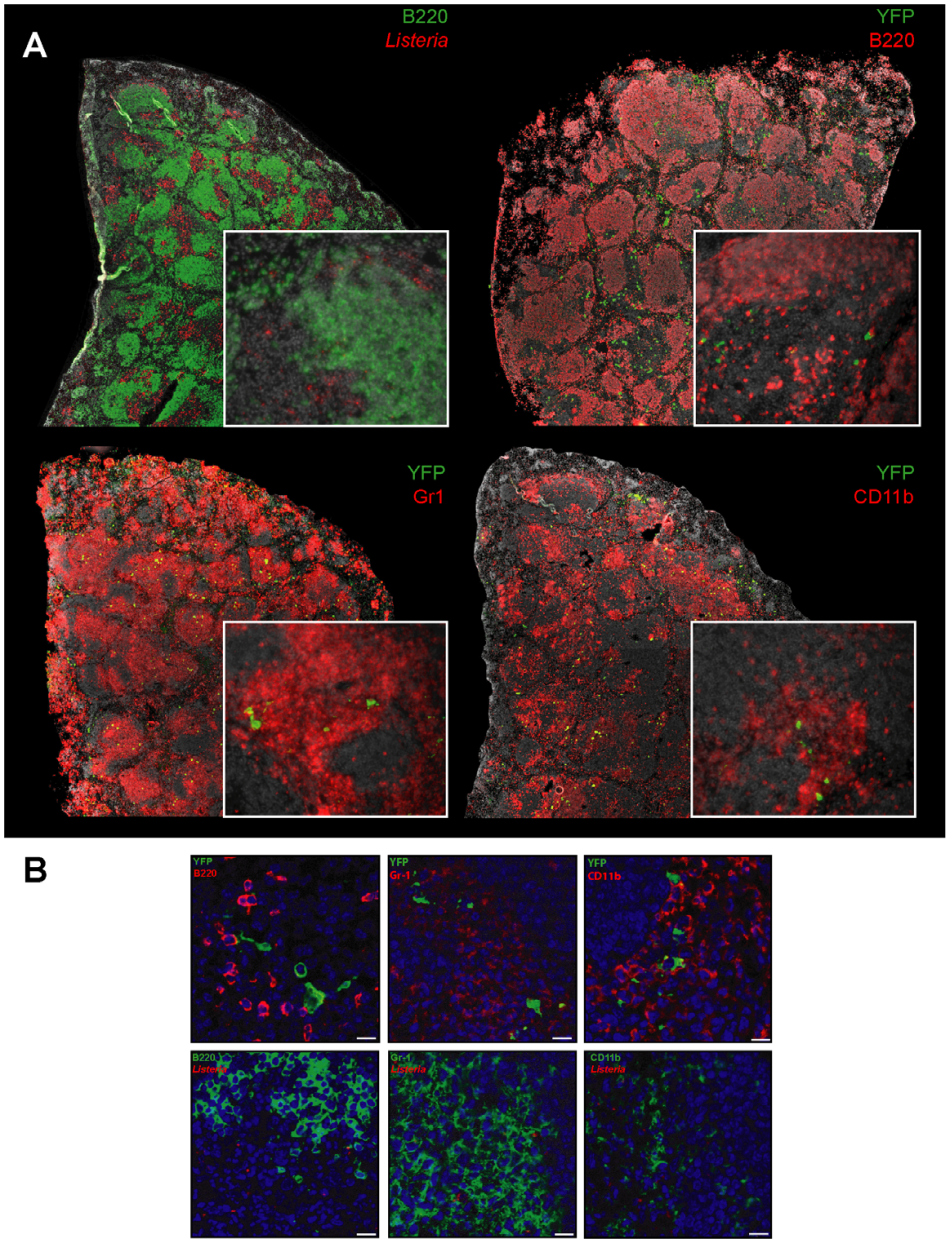
IFNβ/YFP expressing cells are located within the foci of infection in the splenic white pulp. (A) Shown are spleen sections from IFNβ^mob/mob^ mice 24 h after i.v. injection of 10^6^ CFU of *L. monocytogenes*. YFP^+^ cells were detected using a cross reacting polyclonal α-GFP antibody. Signals were amplified with tyramide-FITC for YFP and B220 (shown in green) and tyramide-BIO and Streptavidin-Cy3 for α-*L. monocytogenes*, Gr-1, CD11b, B220 (shown in red). Nuclei in grey stained with DAPI. (B) Presented are high power confocal micrographs of spleen sections from IFNβ^mob/mob^ mice stained as described in (A). Nuclei were stained with DAPI shown in blue. The scale equals 10 µm. The micrographs are representative of at least two independent experiments.

**Figure 4:**
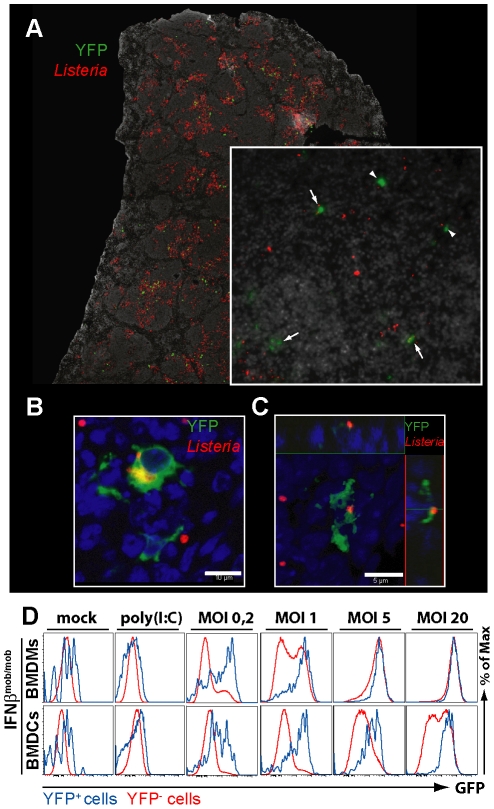
Simultaneous visualization of IFNβ/YFP production and the cellular state of infection after *L. monocytogenes* challenge. (A) Spleen sections from IFNβ^mob/mob^ mice 24 h after i.v. injection of 10^6^ CFU of *L. monocytogenes* were stained as described before. Arrows or arrowheads point at IFNβ/YFP expressing cells that are infected or not infected, respectively. (B)–(C) Confocal micrographs of spleen sections from IFNβ^mob/mob^ mice were prepared as described before. Nuclei were stained with DAPI shown in blue. (C) Shown is an orthogonal section of a z-stack series with the main x/y plane presented as a maximum projection of the z-stack. (D) BMDMs and BMDCs from IFNβ^mob/mob^ and wt mice were generated as described before and were infected with the stated MOIs of GFP expressing *L. monocytogenes*, stimulated with 50 µg/ml poly (I:C) or mock treated for 14 h, respectively,. Extracellular bacteria were killed 1 h p.i. by addition of gentamicin. Cellular IFNβ/YFP production and bacterial load was analyzed by flow cytometry. YFP^+^ (blue line) and YFP^−^ (red line) cells were gated and overlaid in histograms showing their state of infection as determined by GFP. YFP gating was adjusted to the respective wt control (not shown). The experiments shown were repeated twice with similar results.

To visualize the IFNβ/YFP^+^ cells directly in the infected organ we performed fluorescence microscopy of stained spleen sections from *L. monocytogenes* infected IFNβ^mob/mob^ mice (Figure 3). 24 hpi. the majority of infected cells were located within the T cell zone of the splenic white pulp which is consistent with published stainings of *L. monocytogenes* infected spleen sections [21]–[23]. Intriguingly, using the IFNβ^mob/mob^ reporter mouse we were able to show for the first time that the IFNβ/YFP^+^ cells colocalized with larger clusters of CD11b and Gr-1 positive cells in the same splenic compartment (Figure 3A, B). The simultaneous staining for YFP vs. *L. monocytogenes* showed that the majority of the IFNβ^+^ cells were infected with one or more bacteria (Figure 4A–C). As wildtype *L. monocytogenes* are able to escape from the primary cells of infection we used an ActA deficient *L. monocytogenes* strain that is incapable of intercellular spread for a more quantitative analysis of histological sections. This set of experiments revealed that ∼75% of YFP^+^ cells harboured bacteria (data not shown). To investigate if infection with *L. monocytogenes* on a single cell level is a prerequisite for IFNβ production we infected BMDMs and GMCSF-DCs *in vitro* either with *L. monocytogenes* stained with BacLight™ (data not shown) or with GFP expressing *L. monocytogenes* (Figure 4D). The comparison of the state of infection of YFP^+^ cells to that of YFP^−^ cells showed that the YFP^+^ cells carried a much higher bacterial load as the YFP^−^ cells (Figure 4D) indicating that indeed only *L. monocytogenes* infected cells mount an IFNβ response.

These results show that at the peak of the IFNβ response the cytokine producing cells are embedded within the foci of infection in the spleen and are mostly infected with *L. monocytogenes*.

### IFNβ/YFP producing cells are a subpopulation of Tip-DCs

**Figure 5:**
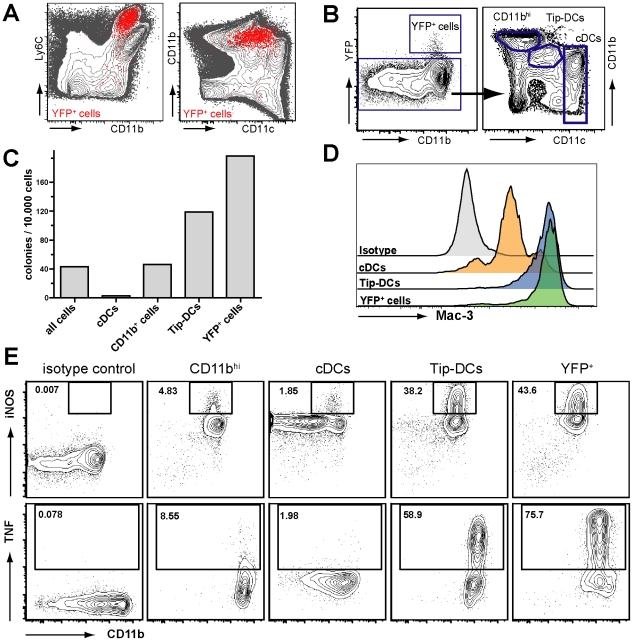
IFNβ/YFP producing cells reveal key features of Tip-DCs after *L. monocytogenes infection*. (A) IFNβ^mob/mob^ mice were i.v. infected with 10^6^ CFU of *L. monocytogenes* for 24 h. The spleens were removed and YFP^+^ cells shown as red dots were analysed in comparison to YFP^−^ cells shown in grey by backgating of flow cytometric data. (B) Gating strategy for FACS sorting of YFP^+^ cells from the spleen against YFP^−^ CD11b^hi^, YFP^−^ *bona fide* TIP-DCs and YFP^−^ cDCs. (C) 10^4^ cells of the given cell populations were sorted into PBS and plated on blood agar plates. After incubation at 37°C for 14 h the bacterial colonies were counted. (D) Sorted cell populations were stained for intracellular Mac-3 and analyzed by flow cytometry. The distributions of Mac-3 expression within the sorted populations are shown as histograms. (E) iNOS and TNF expression from sorted splenic cell populations was determined after intracellular staining via flow cytometry. The gating for iNOS^+^ and TNF^+^ cells was adjusted to the particular staining of cDCs. Ig matched isotype controls were used on sorted total splenocytes. The data shown is representative of at least two independent experiments with spleens from at least two mice pooled for FACS sorting.

FACS analysis for the expression of the myeloid cell lineage markers Ly6C, CD11b and CD11c on IFNβ producing cells in the spleen after *L. monocytogenes* infection revealed that the YFP^+^ cells express these markers in levels earlier described for a specialized DC cell subpopulation called TNF and iNOS producing DCs (Tip-DCs) (Figure 5A) [16], [24]. Tip-DCs are known to be the main producers of TNF and iNOS during *L. monocytogenes* infection and to bear a high intracellular amount of the glycoprotein Mac-3 and variable amounts of the macrophage lineage marker F4/80 [16], [25], [26]. To confirm the hypothesis that the observed IFNβ producing cells were indeed a subpopulation of Tip-DCs we performed FACS sorting experiments *ex vivo* from *L. monocytogenes* infected mice and purified the YFP producing cell population from the spleen alongside with *bona fide* Tip-DCs, cDCs and CD11b^hi^ macrophages, which were all YFP^−^ (Figure 5B). First we determined the bacterial load by plating a defined number of FACS sorted cells onto blood agar plates (Figure 5C). Surprisingly, we found in our approach not the CD11b^+^ macrophages being the mainly infected cells as described earlier [22], [27] but rather the Tip-DCs and the sorted YFP^+^ cells carrying the highest bacterial load. Next we compared the Mac-3 expression of YFP^-^ cDCs, YFP^−^ Tip-DCs, and the YFP^+^ cell population and determined their morphologic characteristics by performing cytospins. While most cDCs showed intermediate expression of Mac-3 the IFNβ/YFP^+^ cells showed a high intracellular expression of Mac-3 comparable to that of Tip-DCs (Figure 5D). On the morphological level cDCs, YFP^+^ cells and the sorted Tip-DCs were indistinguishable from each other (data not shown). As the hallmark feature of Tip-DCs is the secretion of TNF and expression of iNOS we determined these conceptual skills of the sorted cell populations (Figure 5E). Strikingly, high frequencies of iNOS^+^ and TNF^+^ cells were exclusively detectable in the Tip-DC and IFNβ/YFP^+^ cell sample with the YFP^+^ cells showing even the highest frequencies of both iNOS and TNF producing cells (Figure 5E). In contrast to this, CD11b^hi^ macrophages and cDCs show 24 h after *L. monocytogenes* infection only low frequencies of TNF and iNOS producing cells, clearly separating the identity of the IFNβ producers from that of macrophages and cDCs. Histological staining of sequential spleen sections for iNOS and IFNβ/YFP showed that the iNOS^+^ cells colocalized in the same morphologic areas as the YFP^+^ cells in the white pulp of the spleen (Figure S4). These results reveal that the IFNβ producing cells show the exact phenotypical and functional characteristics published for Tip-DCs and thus can be defined as a highly specialized subpopulation of this cell type with IFNβ production as an additional effector function.

### IFNβ^+^ Tip-DCs are T cell priming APCs

**Figure 6:**
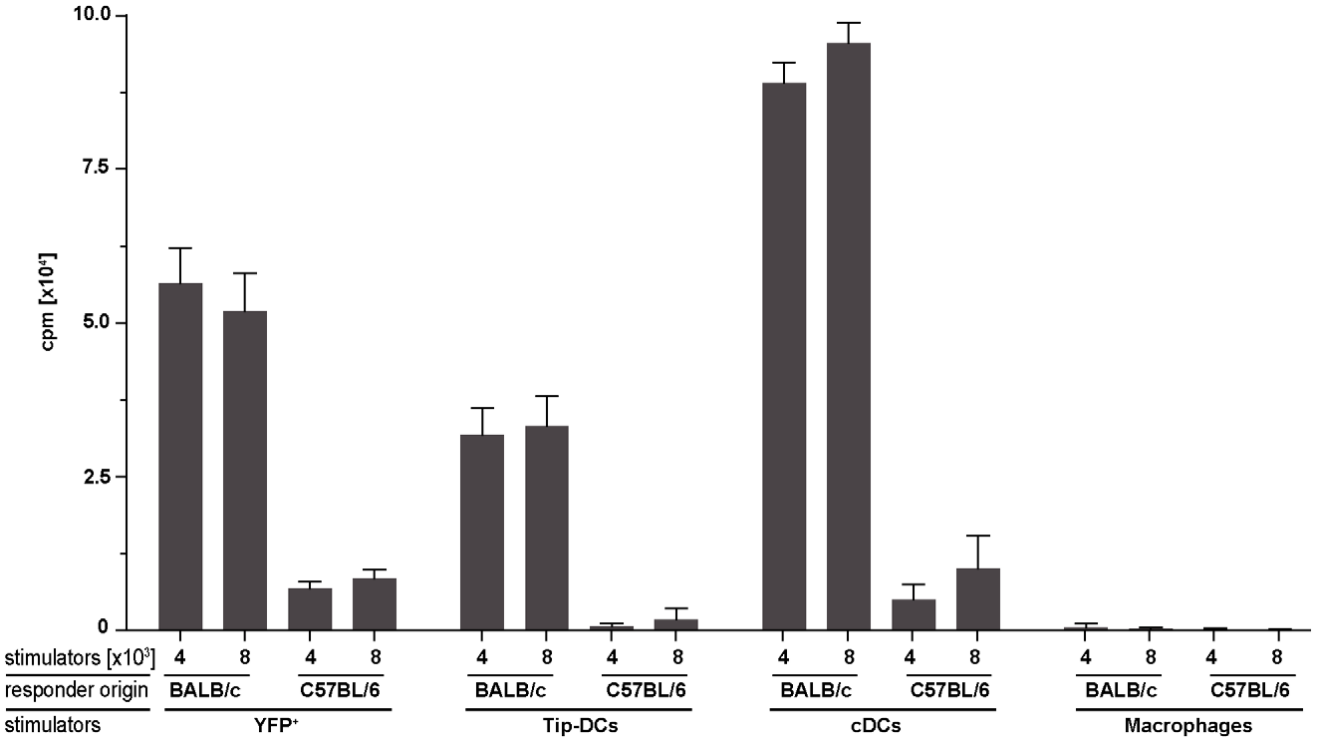
IFNβ^+^ cells show T cell priming capacities. YFP^+^, Tip-DCs, cDCs and macrophages were sorted out of spleens from IFNβ^mob/mob^ mice (C57BL/6) 24 h after infection with 10^6^ CFU of *L. monocytogenes* as shown before. After irradiation with 3000 rad these cells were used as stimulators in an allogenic MLR. Proliferation of CD4^+^ CD62L^+^ naive responder T cells from allogenic BALB/c and isogenic C57BL/6 responder mice in response to the sorted cell subsets was measured by the incorporation of ^3^H-Thymidine. The data is representative for two independent experiments with cells originating from at least three mice per group.

In line with recent cell lineage studies, the surface marker phenotype of the IFNβ producing cells in *L. monocytogenes* infection (Ly6C^hi^, CD11b^+^, F4/80^+^) places these cells within the group of inflammatory monocytes [12], [26]. However, according to our intracellular stainings of effector molecules these YFP/IFNβ^+^ cells are a subpopulation of TNF and iNOS producing DCs. Since the IFNβ^+^ Tip-DCs after *L. monocytogenes* infection were positive for both MHC class II and co-stimulatory markers we interrogated to what extend these cells are bona fide DC. On a functional level the hallmark feature of DCs is the ability to prime naïve T cells [28],[29]. Therefore, we used IFNβ/YFP^+^ Tip-DCs, macrophages, cDCs and *bona fide* Tip-DCs *ex* vivo sorted from *L. monocytogenes* infected spleens as stimulators in a mixed lymphocyte reaction (MLR) and determined their ability to promote proliferation of naïve CD4^+^ T cells (Figure 6). In contrast to the sorted macrophages that did not induce significant T cell proliferation the cDCs showed the highest T cell priming capacity. Intriguingly, the sorted IFNβ^+^ Tip-DCs also stimulated the naïve T cells to proliferate in a highly significant manner (p < 0.0001 as compared to macrophages) and more efficiently than the sorted IFNβ/YFP^−^ *bona fide* Tip-DCs (Figure 6). This result indicates that the IFNβ producing Tip-DCs were professional APCs in promoting the proliferation of naïve T cells described for bona fide DCs and clearly separated them from macrophages.

Taking advantage of the direct cellular visualization of IFNβ production we were able to show that the spleen acts as the major dwelling place of IFNβ producing cells with a small subpopulation of cells (∼10^5^ per spleen) being sufficient to provide this cytokine in biologically functional amounts. Here we show for the first time that these cells were located mainly within the foci of infection in the spleen and were mostly *L. monocytogenes* infected. By evaluating their surface marker pattern and expression of iNOS and TNF we could proof that the IFNβ producing cells were a subpopulation of Tip-DCs with T cell priming abilities of professional APCs. This is to our knowledge the first report demonstrating that Tip-DCs are the main source of IFNβ in an *in vivo* bacterial infection model.

## Discussion

In this work we show for the first time directly *in vivo* that the major cellular source of IFNβ in a relevant bacterial infection model such as murine listeriosis is a specialized subpopulation of Tip-DCs. Besides expression of both TNF and iNOS these cells harbour the ability to prime naïve T cells. The IFNβ producers are mostly infected with *L. monocytogenes* and are exclusively located in the foci of bacterial lesions in the splenic white pulp at the peak of infection.

The observed maximum of IFNβ production in the spleen 24 hpi. is consistent with the previously described peak of IFNβ production [9]. Beside the spleen the liver is shown to be a site of bacterial growth during *L. monocytogenes* infection [30], [31]. Surprisingly, we found only few IFNβ producing cells in this organ. However, we cannot rule out that dying hepatocytes lost during the cell preparation may contribute additionally to the cytokine production. In line with few cells producing IFNβ is the finding that in the liver 10 fold less *ifnb* message is detected as compared to the spleen [32]. One explanation for the low frequencies of IFNβ producing cells in the liver are the organ inherent selftolerizing conditions. As the portal venous blood emerging from the small intestine is enriched in LPS and Toll-like receptor (TLR) agonists distinct immune suppressive mechanisms within the liver ensure sustainment of self tolerance [33].

Only if the pathogen is present in the peritoneal cavity, as is the case after an i.p. or oral infection, significant numbers of bacteria can be recovered from the mLNs, while after i.v. *L. monocytogenes* infection bacteria could be detected only sporadically from there [34]. Our finding that IFNβ producing cells were identified in the mLNs exclusively after i.p. administration of the *L. monocytogenes* correlates with the described presence of the pathogen in the respective lymphoid tissue. Again, this points to the infection with *L. monocytogenes* being a prerequisite for IFNβ production on a cellular level.

During listeriosis clusters of monocytes and neutrophils are rapidly formed within 24 hpi. at the foci of infection in the T cell zone of the splenic white pulp. Monocytes recruited to the lesions differentiate into Tip-DCs driven by IFNγ and produce TNF and NO in large quantities [16], [23]. Our data show for the first time that IFNβ producing cells are also embedded within these clusters of myeloid cells and were mostly *L. monocytogenes* infected.

Here we provide strong evidence that the IFNβ/YFP^+^ cells are a specialized subpopulation of Tip-DCs as they show their surface marker pattern as well as their functional attributes with regard to expression of TNF and iNOS and intracellular Mac-3 as determined by FACS analyses and functional profiling of these cells. Tip-DCs were initially identified as an effector APC subpopulation in the course of *L. monocytogenes* infection in the spleen. In these experiments it was shown that Ly6C^hi^ monocytes are recruited to infected lesions in the spleen where they differentiate into Tip-DCs and are essential for containment of the bacterial growth [2], [16], [25], [35]. The assignment of the IFNβ producers to the group of Tip-DCs stands at first glance in contrast to a recent publication were splenic macrophages were shown to be the major IFNβ producing cells in listeriosis [9]. In this approach sorted CD11b^+^, CD11c^−^, PDCA^−^, B220^−^ cells showed the highest *ifnb* mRNA expression and were assumed to be macrophages according to their surface marker phenotype. The advantage of using the IFNβ reporter mouse model described here is that IFNβ production can be monitored directly on the cellular level by means of YFP production which allows for a more detailed phenotypical and functional FACS characterization directly *ex vivo*.

In our FACS sorting experiments the IFNβ producing Tip-DCs proved to be the cell population most strongly infected with *L. monocytogenes*. Although, one report indicated that sorted macrophages and Tip-DCs both carry similar high bacterial burdens the general view is that Tip-DCs during *L. monocytogenes* infection are bystander cells and only a minor part of them are actually infected [2], [16], [27]. These discrepancies could be explained by insufficient stringency in separating macrophages from Tip-DCs and different approaches chosen for determination of the bacterial load. The IFNβ producing cells identified here might represent the small subfraction of *L. monocytogenes* ^+^ Tip-DCs as described by Pamer et al. [16]. In the liver and the mLNs we also identified cells resembling Tip-DCs by the surface phenotype as the major IFNβ expressing cells. But as a result of low absolute cell numbers of YFP^+^ cells we could not formally proof if these cell fulfill the functional criteria of iNOS and TNF expression, to assign them into the group of Tip-DCs.

A subject of current debate is whether Tip-DCs belong to a specialized group of Ly6C^+^ inflammatory monocyte derived DCs or rather resemble classically activated macrophages. Due to the plasticity of monocyte derived cells under inflammatory conditions it is often difficult to infer from the cell surface marker distribution on the lineage derivation. In particular, besides DCs also monocytes and classically activated macrophages under certain inflammatory conditions show expression of CD11c, upregulate MHC class II and can induce T cell proliferation [13], [26], [36], [37]. By analyzing multiple phenotypical and functional qualities of the IFNβ producers and comparing them to sorted bona fide CD11c^−^ CD11b^+^ macrophages and CD11c^hi^ cDCs we show that they are distinct from *bona fide* macrophages and cDCs: IFNβ/YFP^+^ cells produce iNOS and TNF in higher amounts than macrophages and express higher levels of intracellular Mac-3 than DCs. While in our system sorted macrophages were incapable of significantly inducing naive T cell proliferation the IFNβ producers were able to prime naïve T cells in an MLR albeit less efficiently than cDCs, In this respect the IFNβ producers described here show all the conceptual skills of Tip-DCs as initially introduced by Serbina *et al* [16] and are equipped with features of professional APCs. The developmental relationship of this IFNβ producing Tip-DC subpopulation to other monocytes or monocyte derived cells remains still to be elucidated in detail.

As Tip-DCs are innate effector cells whose main function is to produce cytokines rather than to prime adaptive immune responses the different T cell priming capacities may reflect a distinct mode of operation of cDCs and Tip-DCs.

Tip-DCs are essential for the early containment of bacterial growth as mice lacking CCR2, crucial for Tip-DCs recruiting, or mice deficient for iNOS, TNF or their cognate receptors show increased susceptibility to *L. monocytogenes* infection [38]–[42]. Therefore these cells constitute a first line of defense, keep the pathogen in at bay and shape the initial local cellular and cytokine microenvironment for the adaptive immune system to finally eradicate the pathogen. Since in our experiments the Tip-DCs turned out to be the most strongly infected cell population and the IFNβ^+^ Tip-DCs show a higher capacity to produce TNF and iNOS one could speculate that these IFNβ secreting cells are the terminally differentiated most potent cytokine producing innate immune effector cells. In contrast, it was shown that type I IFNs are detrimental to the host during listeriosis and IRF3^−/−^, IFNβ^−/−^ or IFNAR^−/−^ mice show an enhanced resistance to *L. monocytogenes* challenge [6]–[9]. More specifically, IFNβ deficient mice show a significant decrease in serum IFNα levels proving that the initial production of IFNβ is a prerequisite for high level type I IFN secretion in this infection model. Thus the exclusive lack of IFNβ is already sufficient to render mice more resistant during listeriosis leading to a significantly lower bacterial burden in infected organs and an elevated survival rate as compared to wildtype mice [9]. As a positive impact from type I IFN induction is more the rule than the exception in the course of viral and bacterial infections [43]–[45], the immune system might have evolved to accept the consequences of the fewer cases where they have a detrimental impact [46]–[48]. Pattern recognition receptors as innate surveillance sensors detect many different threats via similar molecular patterns. In this way the innate immune system has only limited possibilities to induce an appropriate type I IFN response after encounter of cytoplasmic bacteria. The induction of IFNβ could be the archetypic response of this yet to be defined receptor.

An alternative explanation would be that the unambiguously beneficial IFN I response in viral infections is hijacked by the *L. monocytogenes* and that by targeting Tip-DCs they trigger IFN I production. They redirect an antilisterial cytokine program by down regulating IFNγ responsiveness [11]. It remains to be proven that the underlying reason for the positive impact of type I IFN production in other bacterial infections is due to other cell types than Tip-DCs being infected by the pathogen.

Taken together Tip-DCs might resemble a double edged sword in the course of an infection with *L. monocytogenes* in that they provide the benefits of iNOS and TNF as well the adverse effects of type I IFN expression.

## Materials and Methods

### Ethics Statement

This study was carried out in strict accordance with the German act for animal welfare (Tierschutzgesetz) § 8. The protocol was approved by the board for nature, environment and consumer protection (Permit number 8.87–50.10.34.08.330) of the regional government of Düsseldorf (North Rhine-Westphalia, Germany). All efforts were made to minimize suffering.

### Mice and Infections

IFNβ^mob/mob^ mice were generated as described previously [17] and were backcrossed for at least 10 generations onto C57BL/6 background. All mice were housed under specific pathogen-free conditions in the animal research facility of the University of Düsseldorf. *L. monocytogenes* infection experiments were performed with *L. monocytogenes* strain EGD, Δ*hly* and Δ*actA L. monocytogenes* (all gifts from M. Hornef, Hannover) and a constitutive GFP expressing *L. monocytogenes* EGD strain (gift from K. Pfeffer, Düsseldorf).

For infection experiments the indicated *L. monocytogenes* strains were grown at 37°C to logarithmic growth phase in Brain Heart Infusion (BHI) medium. The bacteria concentration was determined by OD_600_ measurements and confirmed by determination of the CFU from a serial dilution assay on blood agar plates. The bacteria were washed twice with PBS and were diluted in PBS for infection. For *in vivo* experiments a total volume of 200 µl was injected into the lateral tail vein or the peritoneal cavity of the indicated mice. For *in vitro* infection experiments indicated MOIs were added to replated cells and extracellular bacteria were killed after 1 h by addition of gentamicin (20 µg/ml). For *in vivo* i.v. infection we injected 10^6^ bacteria for 12 and 24 h, 10^5^ for 48 h, and 10^4^ bacteria for 72 and 96 h, respectively. For i.p. infection we used 10^7^ bacteria for 12 and 24 h and 10^6^ *L. monocytogenes* for 48, 72 and 96 h, respectively.

### Antibodies

We used anti CD11b (M1/70), B220 (Ra3-6B2), CD11c (HL3), CD3ε (145-2C11), CD19 (1D3), CD40 (3/23), CD80 (16.10A1), CD86 (GL1), CD8α (53–6.7), Mac-3 (M3/84), Gr1 (RB6-8C5), CD69 (H1.2F3), CD16/32 (2.4G2), Ly6C (AL-21), NK1.1 (PK136) all from BD Biosciences. Anti F4/80 (BM8), Ly6G (1A8) were from BioLegend. Anti GFP and biotin conjugated anti *L. monocytogenes* were from abcam. Anti TNFα (MP6-XT22) was purchased from ebioscience and anti NOS2 for ICS or histology was ordered from Santa Cruz (M-19) or was a gift from Karl Lang (University of Düsseldorf), respectively. Cy3 conjugated Streptavidin was from Caltag, Biotin conjugated donkey anti rabbit, PE conjugated goat anti rabbit, as well as normal sera from mouse; rat, donkey and goat were purchased from Jackson Immuno Research. Isotype matched control antibodies were purchased from BD Biosciences or SantaCruz.

### Generation of mouse BMDMs and BMDCs

For generation of BMDMs, BM cells were cultured in complete (10% [v/v] FCS, 50 µM 2-mercaptoethanol) VLE RPMI 1640 medium (Biochrom) supplemented with 20% L929 cell conditioned supernatant for 6–7 d with an exchange of 50% of culture medium after 3 d. For GM-CSF BMDCs BM cells were cultured in complete VLE DMEM medium (Biochrom) for 8–9 d in the presence of 1,5% GM-CSF containing supernatant from B16 cells with fresh medium added after 3 d and 50% of culture medium replenished after 6 d. pDCs were generated by culturing BM cells in complete VLE RPMI 1640 medium supplemented with 100 ng/ml murine rFlt-3L (R&D Systems) and 10 mM HEPES (GIBCO) for 9 d. 50% of the culture medium was replenished after 5 d.

### FACS Analysis

Organs were digested with collagenase VIII (Roche) and DNase I (Sigma) and stained as indicated. For analysis of liver cells, mice were ketamine/xylazine anaesthetized and subsequently perfused over the left heart ventricle with heparinized PBS (10 units/ml). After digestion the leukocytes were purified via centrifugation at 360 g in PBS/35% percoll. Cells were analyzed for expression of YFP and coexpression of surface markers as indicated. Intracellular staining was performed with the Cytofix/Cytoperm Kit (BD Biosciences) in accordance to manufacturer's reference. For intracellular TNF staining splenocytes were cultured for 4 h at 37°C in complete VLE RPMI 1640 medium supplemented with 1 µl/ml Golgi-plug (BD Biosciences) before subsequent procedures. Samples were analyzed on a FACS Canto II flow cytometer (Beckon Dickinson). FACS sorting was done after MACS depletion of CD3ε^+^ and CD19^+^ cells using a FACS Aria cell sorter (Beckon Dickinson). Flow cytometric data was analyzed with FlowJo (Tree Star).

### Histology

Organs were fixed with 4% PFA for 2–3 h, incubated in 30% sucrose/PBS over night, and frozen in tissue-tek (Sakura). After blocking endogenous peroxidase and biotin 7 µm sections were stained for GFP as described before [17]. Rabbit anti iNOS was stained using a biotinylated anti rabbit antibody. Other indicated antibodies were directly biotin conjugated. The fluorescence signal was amplified with TSA fluorescein or biotin kits (PerkinElmer) according to the manufacturer's instructions. Sections were mounted with Vectashield containing DAPI. Imaging of stained sections was performed on a Nikon Eclipse TE2000E microscope equipped with a Roper Scientific CoolSNAP CCD camera or a Zeiss LSM 510 microscope. Photoshop (Adobe System) was used to select and enlarge YFP^+^ and *L. monocytogenes* ^+^ signals in the splenic overviews and to overlay the images.

### Mixed Lymphocyte Reaction

CD4^+^, CD62L^+^ naïve responder T cells from peripheral LNs of isogenic C57BL/6 or allogenic BALB/c mice were MACS (Miltenyi Biotech) purified and plated out in at least triplicates in 96 well round-bottom plates in complete VLE RPMI 1640 supplemented with penicillin and streptomycin with 2×10^5^ T cells per well. 24 h after i.v. infection of IFNβ^mob/mob^ mice (C57BL/6) with 10^6^ *L. monocytogenes* YFP^+^ cells and YFP^−^ cell populations were FACS sorted as shown in Figure 5B, γ-irradiation (3000 rad) and were add as stimulators in varying numbers to the T cells. The cells were cultivated for 5 days and pulsed with 1 µCi/well [^3^H]thymidin for the last 18 h of culture. Incorporation of [^3^H]thymidin was determined with a liquid scintillation counter. Two tailed paired t tests were performed using GraphPad Prism Software.

## Supporting Information

Figure S1 **Quantification of YFP^+^ cells and bacterial burden in the spleen after infection with titrated doses of** ***L. monocytogenes*** **.** IFNβ^mob/mob^ mice were infected i.v. with 10^5^, 10^6^ or 10^7^ CFU of *L. monocytogenes* for 24 h. (A) The explanted spleens were homogenised and aliquots from a serial dilution of the homogenates were plated on blood agar plates. After 24–48 h of incubation at 37°C the colony number was determined and the CFU per organ was calculated. (B) Shown are the absolute numbers of YFP^+^ cells per analysed organ as measured by FACS. (C, D) Spleen sections from the infected mice were stained for YFP and *Listeria* as described before. Nuclei were stained with DAPI shown in grey. The total number of cells and bacteria for each microscopic image was calculated using the Adobe Photoshop select color tool and the extended histogram window. Numbers of YFP^+^ cells were determined by directly counting the particular cells in the microscopic images. For each dose of infection 10 fields of view from two independently infected mice were analyzed and cell and bacteria numbers are given as bars with SEM.
(TIF)

Figure S2 **Highly activated cells are accountable for the IFNβ expression in the spleen.** Expression of IFNβ/YFP in the spleen 24 h after i.v. infection of IFNβ^mob/mob^ or wt control mice with 10^6^ CFU of *L. monocytogenes*. The FACS plots shown were electronically gated on CD3ε^−^ CD19^−^ live cells, YFP gating was adjusted to wt stainings. The plots shown is representative of at least two independently performed experiments.
(TIF)

Figure S3 **Characterization of IFNβ/YFP producing cells of BMDMs and BMDCs after** ***in vitro L. monocytogenes*** **infection.** Bone marrow cells of the given genotypes were cultured for 6 days in L929-cell conditioned medium to generate BMDMs (A) or were grown with 100 ng/ml Flt3-L or in GM-CSF conditioned medium for 10 days to generate FLT3-L DCs or GM-CSF DCs, respectively (B). The cells were replated and infected with the stated MOIs (A) or a MOI of 20 (B) of the indicated *L. monocytogenes* strain for 12 h. After 1 h the medium was supplemented with 20 µg/ml gentamicin to kill extracellular bacteria. Shown are FACS plots electronically gated on live cells. The YFP gating was done using equally treated wt cells as reference. The data shown is representative of two independently performed experiments.
(TIF)

Figure S4 **IFNβ/YFP producing cells and iNOS^+^ cells are located within the same splenic compartment.** (A) Construction of the superimposed image from serial micrographs. Arrows present the colour layers originating from the serial stains that were overlaid and merged for the final picture. (B) Serial spleen sections from IFNβ^mob/mob^ mice 24 h after i.v. infection with 10^6^ CFU of *L. monocytogenes* are shown. Sections were stained for YFP, B220 and DAPI and for iNOS, B220 and DAPI, respectively. The two serial sections were aligned according to the B220 and DAPI staining from both sections. To eliminate fuzziness the DAPI and B220 layer from the iNOS, B220, DAPI view was deleted. Signals were amplified with tyramide-FITC for YFP and iNOS and tyramide-BIO and Streptavidin-Cy3 for B220. Nuclei shown in grey stained with DAPI. The experiment was performed independently for two times with similar results.
(TIF)
