# Supplementary figures and images for: A Fluorescence Reporter Model Defines “Tip-DCs” as the Cellular Source of Interferon β in Murine Listeriosis
Source: PLoS One. 2010 Dec 16;5(12):e15567. doi: 10.1371/journal.pone.0015567 (PMC3002951; doi:10.1371/journal.pone.0015567)

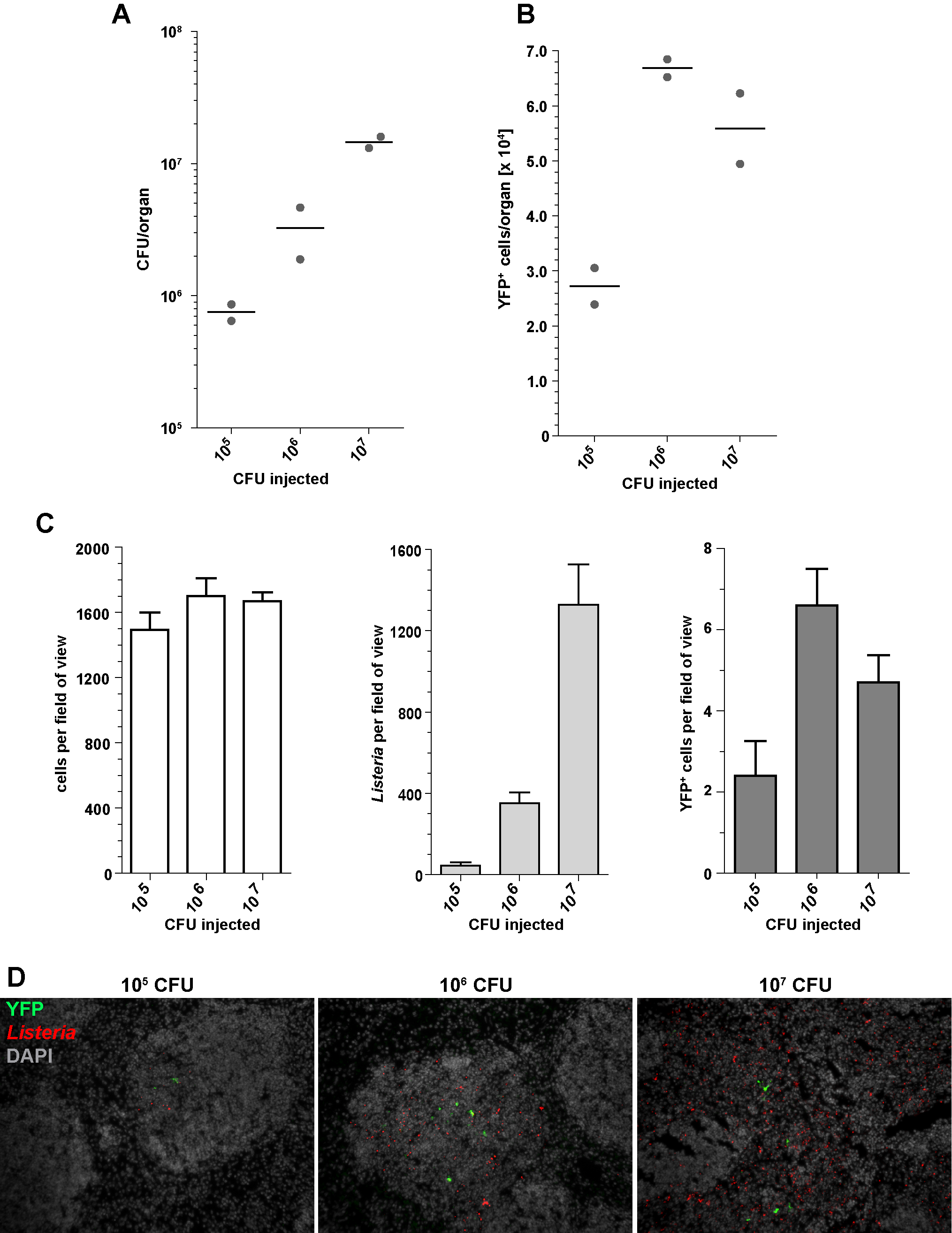

Supplement: Figure S1 — Quantification of YFP+ cells and bacterial burden in the spleen after infection with titrated doses of L. monocytogenes . IFNβmob/mob mice were infected i.v. with 105, 106 or 107 CFU of L. monocytogenes for 24 h. (A) The explanted spleens were homogenised and aliquots from a serial dilution of the homogenates were plated on blood agar plates. After 24–48 h of incubation at 37°C the colony number was determined and the CFU per organ was calculated. (B) Shown are the absolute numbers of YFP+ cells per analysed organ as measured by FACS. (C, D) Spleen sections from the infected mice were stained for YFP and Listeria as described before. Nuclei were stained with DAPI shown in grey. The total number of cells and bacteria for each microscopic image was calculated using the Adobe Photoshop select color tool and the extended histogram window. Numbers of YFP+ cells were determined by directly counting the particular cells in the microscopic images. For each dose of infection 10 fields of view from two independently infected mice were analyzed and cell and bacteria numbers are given as bars with SEM. (TIF) [file pone.0015567.s001.tif]

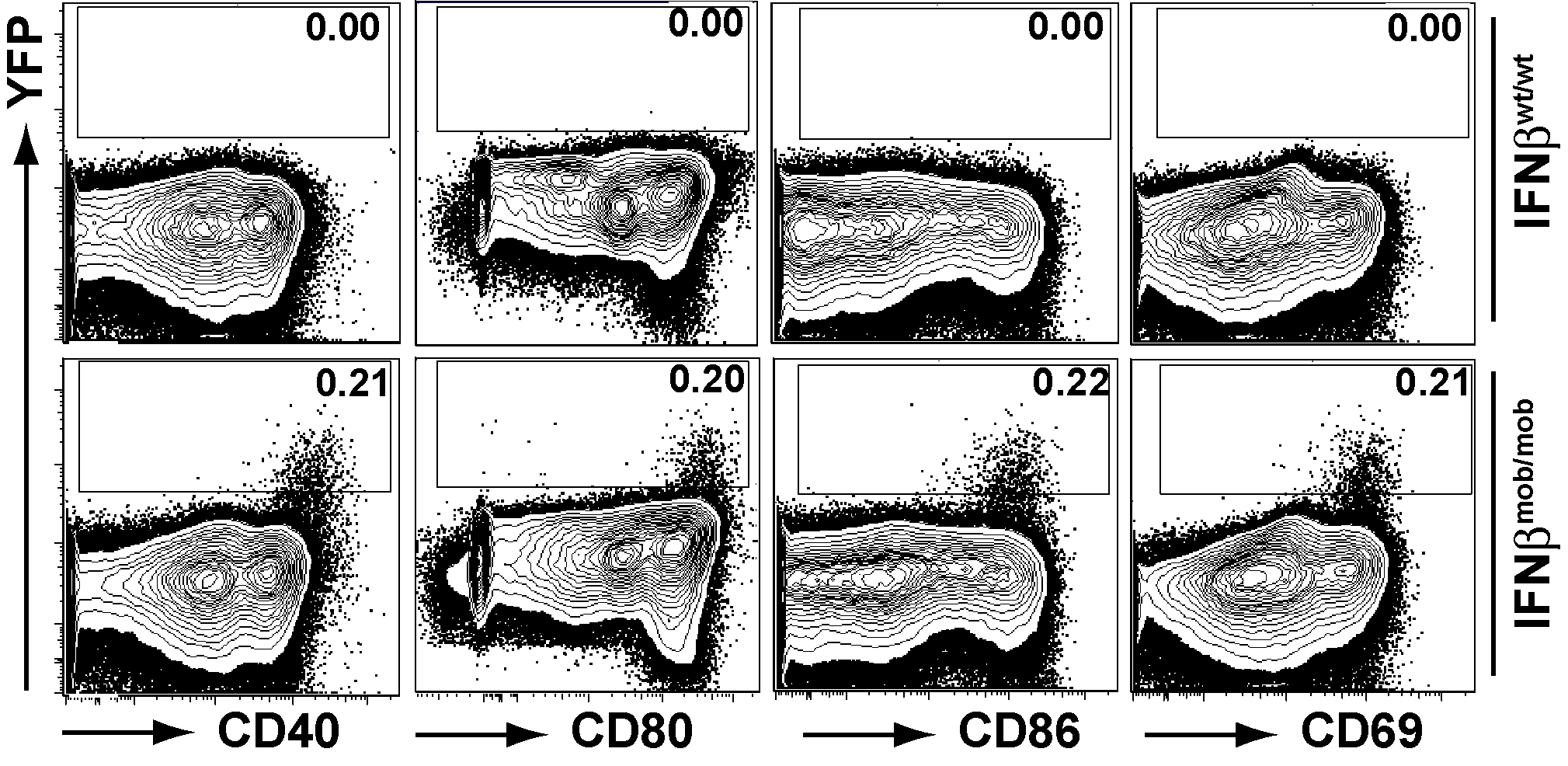

Supplement: Figure S2 — Highly activated cells are accountable for the IFNβ expression in the spleen. Expression of IFNβ/YFP in the spleen 24 h after i.v. infection of IFNβmob/mob or wt control mice with 106 CFU of L. monocytogenes. The FACS plots shown were electronically gated on CD3ε− CD19− live cells, YFP gating was adjusted to wt stainings. The plots shown is representative of at least two independently performed experiments. (TIF) [file pone.0015567.s002.tif]

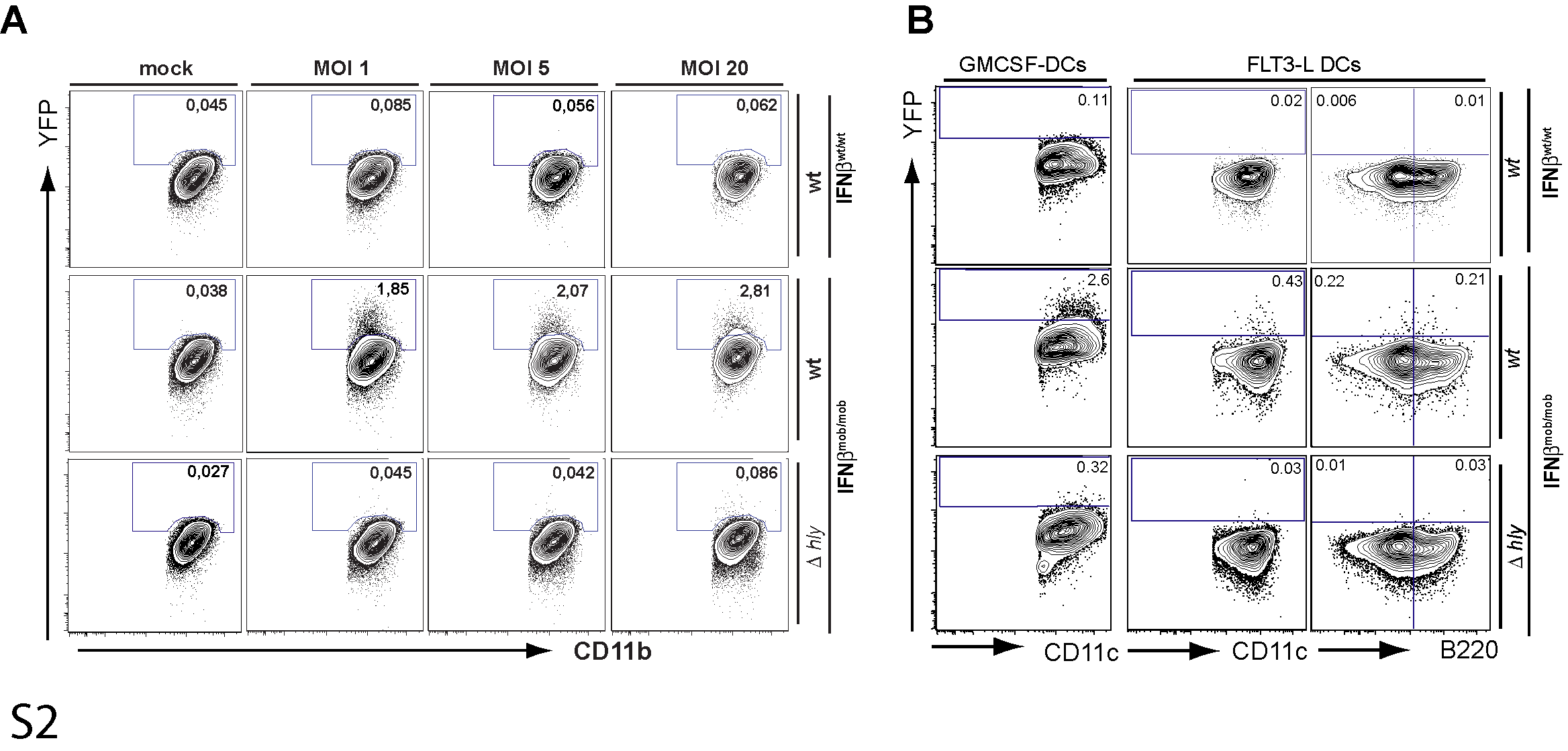

Supplement: Figure S3 — Characterization of IFNβ/YFP producing cells of BMDMs and BMDCs after in vitro L. monocytogenes infection. Bone marrow cells of the given genotypes were cultured for 6 days in L929-cell conditioned medium to generate BMDMs (A) or were grown with 100 ng/ml Flt3-L or in GM-CSF conditioned medium for 10 days to generate FLT3-L DCs or GM-CSF DCs, respectively (B). The cells were replated and infected with the stated MOIs (A) or a MOI of 20 (B) of the indicated L. monocytogenes strain for 12 h. After 1 h the medium was supplemented with 20 µg/ml gentamicin to kill extracellular bacteria. Shown are FACS plots electronically gated on live cells. The YFP gating was done using equally treated wt cells as reference. The data shown is representative of two independently performed experiments. (TIF) [file pone.0015567.s003.tif]

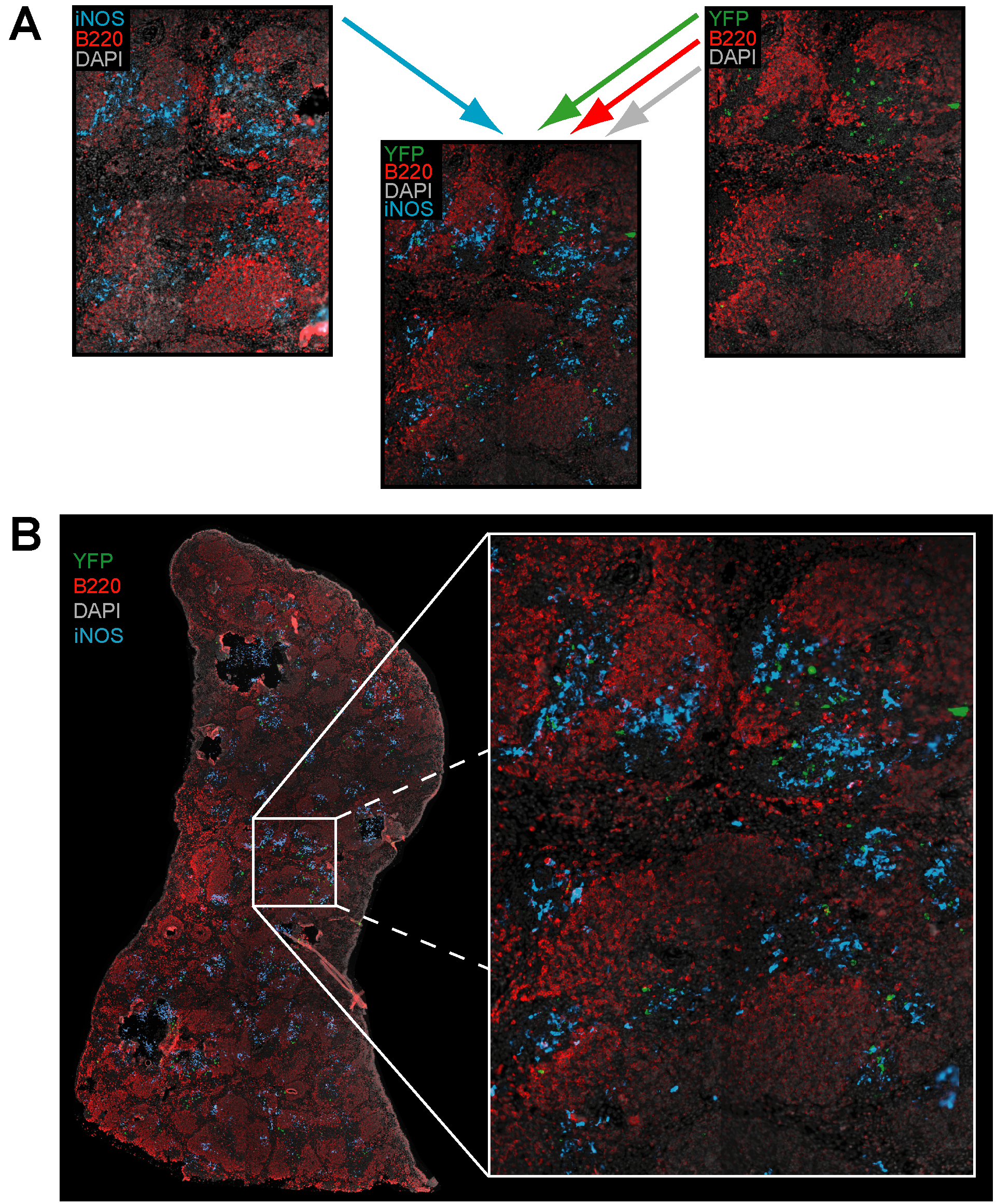

Supplement: Figure S4 — IFNβ/YFP producing cells and iNOS+ cells are located within the same splenic compartment. (A) Construction of the superimposed image from serial micrographs. Arrows present the colour layers originating from the serial stains that were overlaid and merged for the final picture. (B) Serial spleen sections from IFNβmob/mob mice 24 h after i.v. infection with 106 CFU of L. monocytogenes are shown. Sections were stained for YFP, B220 and DAPI and for iNOS, B220 and DAPI, respectively. The two serial sections were aligned according to the B220 and DAPI staining from both sections. To eliminate fuzziness the DAPI and B220 layer from the iNOS, B220, DAPI view was deleted. Signals were amplified with tyramide-FITC for YFP and iNOS and tyramide-BIO and Streptavidin-Cy3 for B220. Nuclei shown in grey stained with DAPI. The experiment was performed independently for two times with similar results. (TIF) [file pone.0015567.s004.tif]
